# MMinte: an application for predicting metabolic interactions among the microbial species in a community

**DOI:** 10.1186/s12859-016-1230-3

**Published:** 2016-09-02

**Authors:** Helena Mendes-Soares, Michael Mundy, Luis Mendes Soares, Nicholas Chia

**Affiliations:** 1 Microbiome Program, Center for Individualized Medicine, Mayo Clinic, 200 First St. SW, Rochester, 55905 MN USA; 2 Department of Surgery, Mayo Clinic, Rochester, MN USA; 3 Harvard Medical School, Boston, MA USA; 4 Department of Physiology and Biomedical Engineering, Mayo College, Rochester, MN USA

**Keywords:** Metabolic network reconstruction, Network, Microbiome, 16S rDNA, Predictive community modeling

## Abstract

**Background:**

The explosive growth of microbiome research has yielded great quantities of data. These data provide us with many answers, but raise just as many questions. 16S rDNA—the backbone of microbiome analyses—allows us to assess α-diversity, β-diversity, and microbe-microbe associations, which characterize the overall properties of an ecosystem. However, we are still unable to use 16S rDNA data to directly assess the microbe-microbe and microbe-environment interactions that determine the broader ecology of that system. Thus, properties such as competition, cooperation, and nutrient conditions remain insufficiently analyzed. Here, we apply predictive community metabolic models of microbes identified with 16S rDNA data to probe the ecology of microbial communities.

**Results:**

We developed a methodology for the large-scale assessment of microbial metabolic interactions (MMinte) from 16S rDNA data. MMinte assesses the relative growth rates of interacting pairs of organisms within a community metabolic network and whether that interaction has a positive or negative effect. Moreover, MMinte’s simulations take into account the nutritional environment, which plays a strong role in determining the metabolism of individual microbes. We present two case studies that demonstrate the utility of this software. In the first, we show how diet influences the nature of the microbe-microbe interactions. In the second, we use MMinte’s modular feature set to better understand how the growth of *Desulfovibrio piger* is affected by, and affects the growth of, other members in a simplified gut community under metabolic conditions suggested to be determinant for their dynamics.

**Conclusion:**

By applying metabolic models to commonly available sequence data, MMinte grants the user insight into the metabolic relationships between microbes, highlighting important features that may relate to ecological stability, susceptibility, and cross-feeding. These relationships are at the foundation of a wide range of ecological questions that impact our ability to understand problems such as microbially-derived toxicity in colon cancer.

**Electronic supplementary material:**

The online version of this article (doi:10.1186/s12859-016-1230-3) contains supplementary material, which is available to authorized users.

## Background

Advances in sequencing technology have culminated in an explosion of 16S rDNA–based microbiome projects, both small [1–3] and large [4–6]. The microbial ecosystems characterized in these projects are the basis for many critical life processes, from global nutrient cycles [7, 8] to homeostasis in the human body [9–11]. The importance of microbiome research is embodied in recent calls for the formation of a worldwide microbiome consortium [12]. The gut microbiome exemplifies a complex system and contains trillions of interacting bacterial cells [13]. It is not sufficient to treat bacterial taxa as independent entities in a statistical framework of association and diversity. Instead, ecological investigation requires examining the biological interactions underlying the complexities of our microbial communities [14].

Efforts to understand complex microbial communities range from inference based on 16S rDNA sequences [15] to the use of ‘omics technologies across multiple time points [16]. A variety of software and tools for analyzing 16S rDNA data exist, and range from identifying taxa [17, 18] and calculating diversity [19] to producing microbe-microbe association networks [20] However, none of these utilize 16S rDNA to understand the mechanistic basis of microbe-microbe interactions. Each measure captures part of a complex picture, but none captures the functional basis [21] for the microbial interactions that make up a community—i.e., the building blocks of the microbiome.

Bridging the gap between association and mechanism in microbe-microbe interactions requires an approach centered on mechanistic principles. One avenue to deciphering the role of a microbe in a community is through the use of a predictive modeling approach [22, 23]. Metabolic models recapitulate the biological processes of nutrient uptake and metabolite secretion [24], which are at the basis of most microbial interactions. Computationally, the reconstruction of genome-scale metabolic models [25, 26] has been automated through large-scale computing efforts such as RAST [27] and ModelSEED [28]. Tools such as COBRA Toolbox [29, 30] provide an interface for manipulating and investigating metabolic network models. Recently, community metabolic models have been generated to explore the gut microbiome in health and disease [31–34], but these efforts have been driven largely by manual curation—a time consuming and laborious practice [26]. Building on these past research efforts, we explore an alternative path to generating predictive community metabolic models for large-scale microbial communities.

The use of metabolic modeling to understand community dynamics is a thriving area of study, as demonstrated by the variety of tools being developed by different groups [35–37]. NetCooperate, for instance, uses a network-based approach to calculate a Metabolic Complementarity Index [38] to predict the metabolic potential for interactions between pairs of organisms [39]. In addition, Zelezniak et al. [40] uses the constraint-based approach to metabolic modeling to infer the types of interactions occurring between species observed to co-occur in nature based on the metabolites predicted to be utilized or secreted by those species.

MMinte (pronounced /‘minti/) is an integrated pipeline that allows users to explore the pairwise interactions (positive or negative) that occur in a microbial network. From an association network and 16S rDNA sequence data, MMinte identifies corresponding genomes, reconstructs metabolic models, estimates growth under specific metabolic conditions, analyzes pairwise interactions, assigns interaction types [41] to network links, and generates the corresponding network of interactions. Our application is composed of a set of seven individual functionalities, known as widgets, that run sequentially, and each widget may also be run as an independent module. We provide a simple example of a mock microbial community. This example can be used for the user to better understand the workflow of MMinte, and make sure MMinte is working as expected by typing ‘Yes’ when asked whether she/he would like to run the example. In addition, we present two case studies from the gut microbiome that illustrate how MMinte can be used to predict ecological features of a microbial community based on metabolic maps of bacterial species. In doing so, MMinte provides a valuable tool for generating well-defined mechanistic hypotheses for further exploration.

## Implementation

MMinte consists of minimally overlapping functions that come together to perform a single task. In designing it, our goal was to facilitate code re-use by focusing on modularity, allowing the user to streamline the parts presented here for other purposes. Indeed, we do not view MMinte as a single-purpose code, but as a set of widgets that can be repurposed for multiple queries, ranging from testing interactions between a set pair of species to reconstructing a community metabolic network. The user is required to provide a file with a measure of association between OTUs in Widget 1 that will define the pairwise interaction that will be analyzed. However, if the user wants to simulate all pairwise analyses between a list of sequences independently of a pre-assumed association, the user can start such an analysis in Widget 2.

The web browser interface creates a point-and-click experience that allows the user to perform complex analysis on large data sets without programming expertise. For those seeking more control or to implement their own pipelines using MMinte widgets, MMinte functions can also be run in a command-line environment. Because all of the code is provided to the user, it can be changed to fit a particular need. Finally, MMinte is under continuous development, it is publicly available on Github (www.github.com/mendessoares/MMinte) for use by the community, and the authors welcome contributions to further its development.

**Fig. 1:**
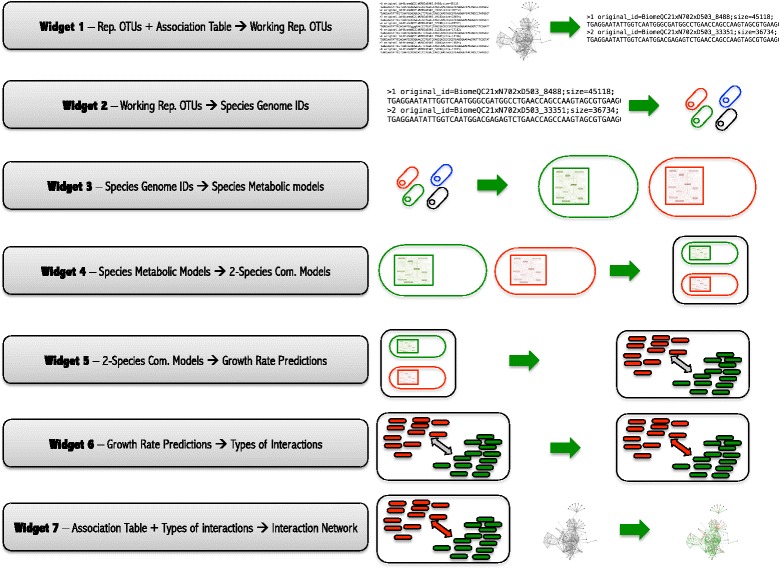
Schematic of the MMinte pipeline. Each green rectangle represents one widget. Widget 1 takes two files, a network of associations between operational taxonomic units (OTUs) and a FASTA file containing the 16S rDNA sequences from a microbiome study, and reduces the latter data set to include only the sequences for OTUs present in the network. Widget 2 identifies the sequences provided and assigns them a genome ID. The percent similarity between the query OTU and the 16S sequence of the genome to which it was matched is stored in a file to be used by Widget 7. Widget 3 calls the ModelSEED service [28] with the list of genome IDs produced by Widget 2, which reconstructs species metabolic models that are exported to the user’s local machine. Widget 4 then uses these species models to create metabolic models for two-species communities. Widget 5 estimates the growth rate of each species in the community under defined metabolic conditions, which can be changed by the user. Widget 6 assesses the types of interaction (mutualism, parasitism, commensalism, competition, amensalism, or neutralism) occurring between the pairs of species in a community based on the effect that each species has on the growth of another. Widget 7 takes the initial information about the topology of the network, the information about the percent similarity between OTUs and the closest genomes, and the types of interactions and plots an interaction network in which the color of the links represents the type of interaction (*positive*, *green; negative*, *red*; *no interaction*, *grey*)

A full run of MMinte generates a predicted network of microbe-microbe interactions for a microbial community using a sequence of seven widgets that progressively analyze 16S rDNA sequences, then genomes, metabolic models, and finally community metabolic networks. The analysis can be run uninterrupted, and all intermediate files are stored. The seven widgets that constitute MMinte are depicted in Fig. 1.

### Widget 1: Reduces data for the downstream analysis

The purpose of this step is to remove operational taxonomic units (OTUs) that will not be used in future analyses, based on an existing list of OTU associations. For plotting of the network in Widget 7, MMinte first creates a network based on the information on the file listing the associations between pairs of OTUs to be analyzed. If pairs of OTUs are not listed in the file as having some level of association, no edges between them are represented. This network is then used as the basis for the depiction of the kind of interactions that are predicted to occur between the OTUs (see description of Widget 7).

#### Inputs

(1) 16S rDNA sequences of all representative OTUs (as one might obtain from, for instance, QIIME [42] or mothur [43]) and (2) an association table between pairs of OTUs.

#### Output

16S rDNA sequences of OTUs.

### Widget 2: Matches 16S rDNA signatures with corresponding genomes

Using BLAST, the 16S rDNA sequences are matched with 16S rDNA sequences from publicly available, complete genomes in NCBI [44]. Importantly, we output a table of percent similarities between OTU- and genome-derived 16S rDNA sequences. If two matches with the same score are found, the first listed is used for further analysis. This information is used to limit potential sources of error from imperfect OTU-genome pairings and to color code nodes in the final network (*Widget 7*).

#### Input

16S rDNA sequences of OTUs.

#### Output

(1) Genome IDs and (2) percent similarity table.

### Widget 3: Obtains metabolic models

This widget uses the web based ModelSEED [28] framework to reconstruct and gap-fill metabolic models under Argonne LB media conditions for a list of genomes.

#### Input

Genome IDs.

#### Output

Single-species metabolic models.

### Widget 4: Merges models

Using COBRApy [45], this function creates metabolic models of two-species communities from a list of pairs of species [33, 46]. The list can be provided by the user or created by MMinte. The merging of the models follows the approach used by [46] for the creation of multi-species stoichiometric models. This approach introduces a fictitious compartment that represents the extracellular environment shared by both species, and adds reactions allowing metabolites that are imported or secreted by each individual species to be transformed into community metabolites.

#### Input

(1) Species-species associations (optional) and (2) single-species metabolic models in the Systems Biology Markup Language (SBML) format.

#### Output

Two-species metabolic models.

### Widget 5: Runs flux balance analysis

This step estimates the growth rates for each species under defined nutrient conditions, in isolation and in the presence of another species, by running a flux balance analysis in COBRApy [45, 47] and follows the procedures of [33]; the algorithm simultaneously maximizes for the biomass objective function of both microbes to estimate the growth rate of each species when in the presence of another organism. The algorithm then silences all reactions of one species while maximizing the biomass objective function of the remaining species to estimate the growth rate of each species individually. This Widget uses a diet file that can be found in the Diets folder. The diet file contains the reactions that determine the availability of 380 metabolites. These are the metabolites present in all the diet files in ModelSEED [28]. In theory, any metabolite can be added to the file and the user is encouraged to do so for metabolites of interest. In the “Complete” diet, the 380 metabolites are available to support the species growth with a flux of import of each metabolite in to the extracellular compartment of 100 mmol/gDW/hr. In “Variant 1” (Complete/10), the flux is ten times slower, and in “Variant 2” (Complete/100), the flux is 100 times slower. The user has the option to specify the nutrient conditions to reflect the specific conditions of the environment being studied.

#### Inputs

(1) Two-species metabolic models and (2) choice of metabolic conditions to be used from media file (provided in the supportFiles folder, default choice = “complete”).

#### Output

Growth-rate predictions.

### Widget 6: Evaluates metabolic interactions

Using previously calculated growth rates, this function quantifies the effect of pairwise interactions and assigns an interaction type to each pair, following Heinken and Thiele [33]. The interactions are determined by their effect on the growth rates of the species when compared to their growth in isolation. When the change in growth rate is over 10%, we consider that an interaction is occurring. The direction is considered negative or positive to the focal species depending on whether the species is predicted to grow slower or faster in a community, respectively. Positive interactions are the ones where at least one species benefits from the interactions and no species suffers from it (mutualism: + +; commensalism: + 0). Negative interaction are interactions where at least one species suffers negative from the interactions (parasitism: + −; amensalsm: − 0; competition: − −). Neutralism represents no interactions between the species (0 0)﻿.

#### Input

Growth-rate predictions.

#### Output

Quantitative effect of interaction and interaction type predicted.

### Widget 7: Draws community metabolic network

This function generates a color-coded interaction network using the D3.js [48] visualization platform, based on the associations provided to Widget 1. Links are colored according to the type of interaction predicted by MMinte (*Widget 6*). The shading of a node reflects the percent similarity between OTUs and genomes (*Widget 2*).

#### Input

(1) Association table between pairs of OTUs, (2) percent similarity table, and (3) quantitative effect of interaction and interaction type predicted.

#### Output

**Fig. 2:**
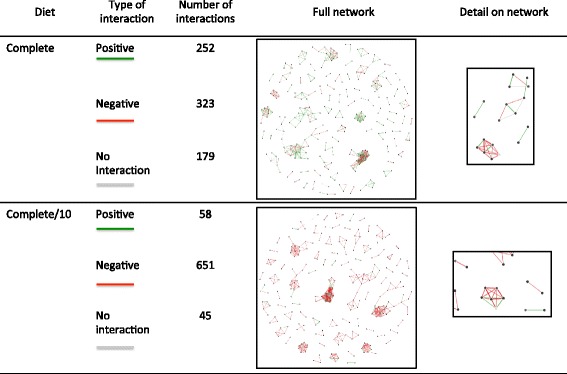
Network and number of the different types of interaction for operational taxonomic units in Case Study 1 under “Complete” and “Complete/10” metabolic conditions. There are 380 metabolites in the “Complete” metabolic conditions and they exist as highly available. The metabolic condition “Complete/10” contains the same metabolites as “Complete” but at 10 times lower availability. Please see file Diet.txt for a complete list of the metabolites, and their availabilities represented as uptake metabolic fluxes. It can be seen from the figure that a 10 fold reduction in metabolite availability resulted in a significant decrease in the number of positive interactions predicted to occur between the members of this community, with parallel increase in the number of negative interactions. MMinte first creates a network based on the information on the file listing the associations between pairs of OTUs to be analyzed. If pairs of OTUs are not listed in the file as having some level of association, no edges between them are represented. This network is then used as the basis for the depiction of the kind of interactions that are predicted to occur between the OTUs. In the network, the shading of the nodes indicates how close the match between the sequence of the OTU is to the sequence of the genome assigned to it. This can be seen in the details from the full networks plotted. The darker the node, the higher the similarity. The length and thickness of the links reflect the association values on the initial file provided by the user. The shorter and thicker the line, the higher the association value. The color of the links reflect the kind of interaction. The red, green and grey represent whether the interaction between the two species is predicted to be negative, positive or no interaction is predicted, respectively

Metabolic interaction network (see Fig. 2).

## Results and discussion

### Usage

Below we present two case studies that exemplify how MMinte can be used to predict microbial interactions under user defined metabolic conditions. The user can choose to run the default example representing a mock community created by the authors as a test for the performance of the software. All files used in these examples, and a full tutorial on how to perform these analyses, can be found in the Additional file 1: Table S1 and in the project folder at github.com/mendessoares/MMinte.

#### Case study 1

With just two user-provided files, one containing correlations between pairs of OTUs and the other representative sequences from a microbiome study, MMinte creates an association network in which the color of the links represents the types of predicted interactions between pairs of OTUs in a microbial community.

In Case Study 1, we use data from the Human Microbiome Project (HMP) to demonstrate MMinte’s potential for exploratory research studies that focus on the dynamics of host-associated microbial communities. The HMP is a multi-institutional collaboration that allowed the description of the microbial diversity of several sites of the human body in healthy individuals. The study was reviewed by each participating institution’s Institutional Review Boards. Full information can be found in [5]. To demonstrate how users can take advantage of this tremendous resource, the data used in this example are a subset of what is available on the HMP page (http://hmpdacc.org/HMQCP/, uncompressed files from rep_set_v13.fna.gz and otu_table_v13.txt.gz) [4, 5], allowing users to rerun the analysis in a straightforward and fast way, while taking advantage of publicly available data. We subset the full data file, out_table_v13.txt, from the HMP database and calculated the Pearson correlations for the OTUs across samples using the R statistical package [49]. The reduced dataset contains 659 associations for 308 OTUs representing 176 species.

##### The problem

The number of positive and negative interactions in a community influences its level of stability and consequently its resistance to invasion by pathogens [50]. With MMinte, we can run our analysis in a variety of metabolic conditions and quantify the number of positive and negative interactions predicted for each. This will generate hypotheses regarding the metabolic conditions likely to favor stability of the system.

##### The results

We ran the full MMinte pipeline by clicking the “run all” tab on MMinte’s introductory page and providing two files, corrs.txt (which contains the associations between OTUs) and seqs.txt (which contains representative sequences) using the default setting of “complete” for the metabolite availability condition, which represents a condition with 380 metabolites available in large amounts. MMinte predicted 252 positive and 323 negative interactions between pairs of OTUs in addition to 179 pairs lacking any type of of interaction (Fig. 2, *left panel*). We then reran Widget 5 with different metabolite availability conditions. Figure 2 (*right panel*) shows the same correlation network with a metabolite availability that is 10 times lower, resulting in different predicted interactions (58 positive and 651 negative interactions between OTUs and 45 pairs lacking any interaction). This result is consistent with the prediction that lower nutrient availability will favor more competition between organisms.

The results of this analysis highlight some possible characteristics of the community that could not be inferred solely from association data. For instance, assuming stability, and thus protection against pathogen invasion, is greater in communities with more competitive interactions [50], then the metabolic conditions that lead to the predicted network shown in Fig. 2 (*right panel*) are likely to promote more stability. In addition, if we assume that stronger positive correlation values between pairs of species indicate positive interactions [51], the network of interactions observed under metabolic conditions equivalent to the ones listed under “complete” are more reflective of the real system than the alternative metabolic conditions tested. These are just two examples of the window microbe-microbe interactions—the building blocks of community networks—provide for understanding their ecology.

#### Case study 2

In the following example, we use data from Rey et al. [52], who investigated the growth of the sulfate-reducing bacterium *Desulfovibrio piger* in the guts of gnotobiotic mice, in the presence of eight other bacterial species and under different nutritional conditions*. D. piger* is the most commonly found sulfate-reducing bacterium in healthy adults, and is thought to shape the responses of the gut microbiota to dietary changes [52]. However, relatively little is still known about the niche this species occupies and how it may influence the metabolism of the other microbial species found in the gut [52].

##### The problem

The interactions between *D. piger* and other members of the gut microbial community have been shown to influence the level of H_2_S in the gut. However, *D. piger* has a variety of potential metabolic pathways, only some of which will lead to the production of H_2_S. The role of interactions in determining the metabolic niche of *D. piger* in the gut is both important and not fully understood. Using MMinte, we explored the types of interactions that are predicted to occur between nine different species of microbes that co-occur in the human gut and whose interactions are believed to be metabolically based [52, 53]. We created a set of metabolic conditions where we varied the availability of oxygen, chondroitin sulfate, and fructose. These represent some of the metabolites that were manipulated in the experiments of Rey et al. [52].

##### Results

We started by providing a list of species IDs to Widget 3 (*D. piger*: model 411464.8, *Bacteroides thetaiotaomicron*: model 226186.12, *Bacteroides caccae*: model 411901.7, *Bacteroides ovatus*: model 28116.7, *Eubacterium rectale*: model 657318.4, *Marvinbryantia formatexigens*: model 478749.5*, Collinsella aerofaciens*: model 411903.6, *Escherichia coli*: model 83333.113, and *Clostridium symbiosium*: model 742740.3). After reconstructing the individual species metabolic models and creating two-species communities (*Widget 4*), we predicted species growth rates in the presence and absence of another species in the community by running Widget 5 under 17 different metabolic conditions, listed in Additional file 1: Table S1. To parallel our analysis in Case Study 1, we also calculated the number of positive and negative interactions under metabolic conditions containing 380 metabolites with different availabilities.

A look at the predicted growth rate of *D. piger* in the presence and absence of other species in the community shows that this species is likely to benefit from the presence of each of the other species in the community under “Complete” metabolic conditions. *D. piger* is consistently predicted to grow under aerobic conditions, but under anaerobic conditions, growth is only predicted to occur if either *B. ovatus*, *B. thetatiotaomicron*, *B. caccae*, *C. symbiosium*, or *E. coli* are present. Thus, using the models reconstructed using ModelSEED, MMinte predicts an obligate association between *D. piger* and these species in anaerobic environments (Additional file 1: Table S1). Interestingly, *D. piger* impaired the growth of most species it was paired with under all conditions except “Complete” (Additional file 1: Table S1). Exceptions were *E. coli* and *E. rectale*; the magnitude of the effect of *D. piger* on their growth depended on the flux conditions for oxygen, chondroitin sulfate, and sulfate (Additional file 1: Table S1). Even though our analysis only focused on variations in three metabolites, the results provide some insight into the niches that these species may occupy and how they are predicted to interact under a variety of metabolic conditions.

**Fig. 3:**
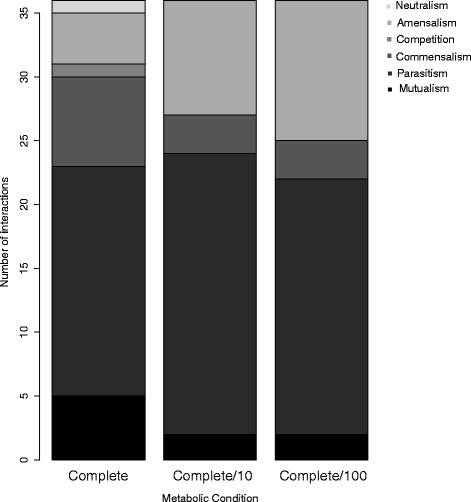
Number of each type of interaction predicted to occur between pairs of the nine bacterial species (*D* *esulfovibrio* *piger*, *Bacteroides thetaiotaomicron*, *Bacteroides caccae*, *Bacteroides ovatus*, *Eubacterium rectale*, *Marvinbryantia formatexigens, Collinsella aerofaciens*, *Escherichia coli*, and *Clostridium symbiosium*) inoculated into the guts of gnotobiotic mice under different metabolic conditions in [52] and used in Case Study 2. The metabolic conditions simulated in MMinte were “Complete”, “Complete/10” and “Complete/100”. There are 380 metabolites in the “Complete” metabolic conditions and they exist as highly available. The metabolic condition “Complete/10” contains the same metabolites as “Complete” but at 10 times lower availability and “Complete/100” contains the same metabolites as “Complete” but at 100 times lower availability. Please see file Diet.txt for a complete list of the metabolites, and their availabilities represented as uptake metabolic fluxes. The effect of being in a community on the growth rate of each species (positive +, negative -, or no effect 0) determines the kind of interaction occurring. The interactions are defined as: mutualism: + +; parasitism: + −; commensalism: + 0; competition: − −; amensalism: − 0; neutralism or no interaction: 0 0)

Overall, the number of each type of interaction changed depending on metabolite availability, but not linearly (Fig. 3). For instance, with a 10-fold decrease in metabolite availability, the number of predicted parasitic interactions increased—but with a further 10-fold decrease in metabolite availability, the number of predicted parasitic interactions then decreased. This suggests that alternative metabolic pathways may be invoked depending on the amount of particular metabolites and not necessarily on their presence or absence, affecting how different organisms interact with each other. These results are in concordance with the observation that the nutrient conditions that organisms experience are predicted to have marked effects on the kinds of interactions they have (Fig. 3).

##### General discussion

MMinte bridges the gap between association and mechanism in microbe-microbe interactions by assessing the metabolic influence that two microbes have on each other. Our predictive modeling approach involves reconstructing the pairwise metabolic community models that make up the basic unit of interaction within a community. More specifically, MMinte advances microbiome research by assigning functional interactions instead of simply calculating associations or correlations based on abundance [51]. This allows us to capture the effect of metabolite exchange on the interactions of an entire microbial community across different nutrient conditions, thus providing an important link to the overall drivers of environmental dynamics.

The metabolic interactions that MMinte identifies can be used to understand the broader ecological features of a biological system. Dynamic ecological features such as stability and robustness are linked to competitive-cooperative interactions and the nature of the positive-negative feedback loops they engender [50]. For example, it has been widely posited that negative interactions self-regulate and stabilize fluctuations within a community [54, 55]. In Case Study 1, MMinte showed that out of 754 total associations detected among a subset of human microbiome species, 33.4% were predicted to be positive and 42.8% negative under “Complete” metabolic conditions. The rest (23.7%) were predicted to not represent significant metabolic interactions between the species. When fasting conditions are modeled by decreasing the availability of metabolites by an order of magnitude, MMinte predicts that 7.7% of interactions will be positive, 86.3% will be negative, and in 6% of the cases, no interactions will occur. This finding intuitively matches the expectation that competition increases in a community with limited nutrient availability [56]. MMinte enables users to grasp these important ecological interactions and better understand the role of competition and cooperation in community stability [57, 58].

Case Study 2 highlights the modular nature of MMinte and the ability of the user to explore the effect that changes in the availability of a particular metabolite may have on the interactions between organisms. The results give us an important window into the role of the environment, specifically the presence or absence of oxygen, chondroitin sulfate, and sulfate, on the interactions between *D. piger* and other organisms commonly found in the gut. *In vivo* experiments have shown that mice colonized solely with *D. piger* have significantly increased levels of H_2_S, which is a genotoxic metabolite that may be involved in the development of colorectal cancer [59, 60], compared to mice colonized with a consortium containing the other eight species analyzed here. Understanding the metabolite conditions favoring the dominance of the other species over *D. piger* can help inform dietary interventions aimed at reducing the abundance of this species in the gastrointestinal tract.

Constraint-based metabolic models were first used to explore the mechanistic bases of interactions between a sulfate-reduced and a methanogen [61, 62]. In this work, the authors created dual-species stoichiometric models that comprised the central metabolism of the bacteria *Desulfovibrio vulgaris* and *Methanococcus maripaludis.* The model accurately predicted the ratio of the two species during cell growth and the flux of several metabolites, thus demonstrating the utility of this modeling approach to understand the mechanistic bases of interactions [61]. Furthermore, the potential associated with using constraint-based analysis to better understand species interactions and explore the properties of communities with more than two species has been shown in previous studies. For instance, Zelezniak et al. [40] were able to infer the types of interactions occurring between species observed to co-occur in nature. The authors reconstructed the GEMs for 261 species from 1297 communities, and for each community calculated the metabolic resource overlap and the metabolic interaction potential by counting the minimal number of components required for the growth of all members considering that they all interacted with each other or that no interactions occurred. The use of a “species metabolic interaction analysis” (SMETANA) score, which estimates the strength of metabolic coupling in the community, then allowed them to have an estimate of the degree of dependency on exchanged metabolites as a proxy for interaction strength. They found that whereas resource competition is apparent in all communities due to habitat filtering, mutualistic interactions are prominent in co-occurring subcommunities. MMinte allows users to perform similar large-scale assessments of pairwise microbial metabolic interactions under a variety of metabolic environments for their community of interest. The results of the analysis can then be used to explore how the interactions between the species affect community features.

Like all algorithms, MMinte has potential limitations. For example, the predictions created by MMinte are only as accurate as the metabolic models used. These metabolic models are linked from 16S in a multi-step process that involves identification of genomes and metabolic network reconstruction using ModelSEED [28]. This automatic reconstruction approach may still be unable to create models with the same quality as those improved by manual curation, and some metabolic capabilities unique to a particular species may not be represented. However, automatic reconstruction allows the creation of a large number of models that reflect a large proportion of the metabolic capabilities of the organisms under study. Furthermore, improved algorithms are being developed that will reconstruct models that more accurately reflect the full metabolic potential of individual species. Missing data in the genome database or in the biochemical database are also both potential sources of error. Conversely, as databases rapidly grow, so will the accuracy of MMinte’s predictions. Another limitation that needs to be acknowledged is that, at this stage, our approach fails to integrate the effect that other species in a community may have on the type and strength of interactions between two particular organisms, as is known to occur [37]. Even so, MMinte helps the user minimize the potential for over-interpretation by visually displaying the percent similarity between 16S rDNA provided by the user and the genomic data,

## Conclusions

MMinte is a tool that predicts the type of interactions occurring between organisms in a complex microbial community under defined metabolite conditions based on the metabolic models of each species. A full run takes data of an association network and 16S rDNA sequences, identifies the genomes, reconstructs metabolic models, and estimates the effect of being in a two-species community for each species under user defined metabolic conditions. The predicted interactions are then plotted in an interaction network. Additionally, the widgets that make up MMinte can be run independently allowing the user to perform specific tasks and bypass some of the steps of the analysis. We have incorporated the design principles of clear modularity, usability, and open access into the development of MMinte. In our view, part of the value of MMinte to the development of predictive community metabolic modeling is the potential for integration into other analytical platforms. We view the ability to build on existing development efforts as critical to expanding systems biology tools to wider and broader scales of ecology and data [14]. MMinte is thus a fundamental tool for exploring a large number of interactions, allowing researchers to move beyond the use of statistical measures of association into biologically relevant analysis of interactions between the species in a microbiome.

## Additional file

Additional file 1: Table S1. (XLSX 78 kb)

## Abbreviations

COBRA: Constraint-based reconstruction and analysis
FBA: Flux balance analysis
MMinte: Microbial metabolic interactions
